# Joint longitudinal data analysis in detecting determinants of CD4 cell count change and adherence to highly active antiretroviral therapy at Felege Hiwot Teaching and Specialized Hospital, North-west Ethiopia (Amhara Region)

**DOI:** 10.1186/s12981-017-0141-3

**Published:** 2017-03-16

**Authors:** Awoke Seyoum, Principal Ndlovu, Zewotir Temesgen

**Affiliations:** 1 Department of Statistics, Bahir Dar University, Bahir Dar, Ethiopia; 2 Department of Statistics, UNISA, Pretoria, South Africa; 3 School of Mathematics, Statistics and Computer Science, University of KwaZulu Natal, Durban, South Africa

**Keywords:** HAART, Multivariate longitudinal data, Joint model, Adherence, CD4 cell count change

## Abstract

**Background:**

Adherence and CD4 cell count change measure the progression of the disease in HIV patients after the commencement of HAART. Lack of information about associated factors on adherence to HAART and CD4 cell count reduction is a challenge for the improvement of cells in HIV positive adults. The main objective of adopting joint modeling was to compare separate and joint models of longitudinal repeated measures in identifying long-term predictors of the two longitudinal outcomes: CD4 cell count and adherence to HAART.

**Methods:**

A longitudinal retrospective cohort study was conducted to examine the joint predictors of CD4 cell count change and adherence to HAART among HIV adult patients enrolled in the first 10 months of the year 2008 and followed-up to June 2012. Joint model was employed to determine joint predictors of two longitudinal response variables over time. Furthermore, the generalized linear mixed effect model had been used for specification of the marginal distribution, conditional to correlated random effect.

**Results:**

A total of 792 adult HIV patients were studied to analyze the longitudinal joint model study. The result from this investigation revealed that age, weight, baseline CD4 cell count, ownership of cell phone, visiting times, marital status, residence area and level of disclosure of the disease to family members had significantly affected both outcomes. From the two-way interactions, time * owner of cell phone, time * sex, age * sex, age * level of education as well as time * level of education were significant for CD4 cell count change in the longitudinal data analysis. The multivariate joint model with linear predictor indicates that CD4 cell count change was positively correlated (p ≤ 0.0001) with adherence to HAART. Hence, as adherence to HAART increased, CD4 cell count also increased; and those patients who had significant CD4 cell count change at each visiting time had been encouraged to be good adherents.

**Conclusion:**

Joint model analysis was more parsimonious as compared to separate analysis, as it reduces type I error and subject-specific analysis improved its model fit. The joint model operates multivariate analysis simultaneously; and it has great power in parameter estimation. Developing joint model helps validate the observed correlation between the outcomes that have emerged from the association of intercepts. There should be a special attention and intervention for HIV positive adults, especially for those who had poor adherence and with low CD4 cell count change. The intervention may be important for pre-treatment counseling and awareness creation. The study also identified a group of patients who were with maximum risk of CD4 cell count change. It is suggested that this group of patients needs high intervention for counseling.

## Background

Sub-Saharan Africa has the most serious HIV and AIDs epidemic in the world. In 2013, approximately 24.7 million people were living with HIV, accounting for 71% of the global total. In the same year, there were around 1.5 million new HIV infections and 1.1 million AIDs-related deaths. The 2013 report indicates that, in sub-Saharan African countries, the prevalence rate among both female and male sex workers was still high (13%) [1]. Ethiopia, as one of these countries has been affected by the epidemic with a prevalence of 1.5% [2]. Its burden has been high in the Amhara Region, including the catchment area of Felege Hiwot Teaching and Specialized Hospital [3]. Because of its large population size, the Amhara region has the largest Prevalence of human immunodeficiency virus [3]. Although the HIV prevalence shows a decreasing rate, still it bears a significant proportion of the epidemic burden as compared to the national and worldwide encumbrance [4]. HIV affects the CD4 cell count in the human body, so it can be employed to make appropriate decisions for the initiation of HAART and proper management of the progression of the infection [5, 6]. Patients’ CD4 cell count should recover/rebound/at least to the lower limit of the CD4 cell count for the general healthy adult population (500 cells/mm^3^), which, otherwise, can be an indication of immunologic failure [7].

In addition to HIV, there are also other factors affecting CD4 cell count changes. Some of these are demographic variables such as age (older ages are predictors of lower count response to HAART) [7, 8], sex (females experience better CD4 count response to HAART compared to males) [9, 10], and residence area (rural patients who start ART with a deteriorated CD4 cell count at the initiation of HAART poorly respond to HAART [11]. Previous studies also indicate that WHO’s clinical stage is an independent predictor of CD4 cell count at enrolment [12]. Other investigators state that there is a positive association between baseline CD4 cell count and its size after initiation of treatment [13, 14]; however, this result was inconsistent with the result of other studies [8]. Identifying factors which influence the level of CD4 cell count other than HAART helps health professionals and patients to facilitate proper management and monitoring of health care interventions to be effective. Moreover, it helps check whether or not HIV patients who initiate HAART with baseline CD4 cell count ≤200 cells/mm^3^ recover to the normal range of CD4 cell count after commencement of HAART [7].

On the other hand, the principal factors associated with non-adherence appear to be patient-related factors including substance and alcohol abuse [15]. However, other factors such as inconvenient dosing frequency, dietary restrictions, pill burden and side-effects, poor patient–care service provider relationships and poor care service provision may also contribute to the failure of adherence [16]. Another study disclosed that different factors such as sex, residence area, and ownership of cell phone contributed to patents’ irregular use of medications [17]. Another investigation indicated that patients’ current substance use concerning HAART, their beliefs about the necessity of HAART, and their trust in the HIV care provider are determinants for their non-adherence [18].

In HAART treatment, the number of CD4 cell count and adherence to HAART are measured repeatedly over time [15]. Patients’ improvement of adherence to HAART provides progression of CD4 cell count in the blood of HIV patients; and this increment of CD4 cell count at every visiting time encourages the patient to be a good adherent to HAART [19]. Hence, the above findings indicated that there are discrepancies among the findings of different studies. This discrepancy of findings suggests that identifying and overcoming the factors that reduce adherence to antiretroviral agents is of utmost importance for prolonged CD4 cell count progression and viral load suppression [15].

A number of key issues are raised in the study of adherence to antiretroviral therapy and CD4 cell count change, determinants that affect jointly, and the development of interventions. Addressing these issues may provide valuable information about which patients are most at risk for non-adherence and about how adherence can be improved. It is well known that non-adherence in the treatment of HIV compromises the effectiveness of therapy or progression of CD4 cell count change [20].

In many practical situations, we observed two or more longitudinal outcomes that need joint modeling of the response variables to identify predictors that affected jointly. Joint models are used to describe the joint behavior of the two response variables simultaneously. These responses may have varied characteristics; that is, they might be binary, ordinal or continuous in nature.

Many previous studies had employed joint models of longitudinal outcomes and time to event [21–24]. Many of these methods were used for the joint modeling of longitudinal data and survival process outcomes, which have typically allowed a univariate outcome and a solitary event time. These studies did not consider two longitudinal values observed repeatedly from the same subject and lacked multivariate analysis of two repeatedly observed results. Joint modeling between two longitudinal outcome variables has advantages in reducing type I error rates in multiple tests with repeated observation on the same subject and improves efficiency in estimating parameters [25]. Thus, the current study was conducted with the objective of reviewing Generalized Linear Mixed Model (GLMM) approach that can be extended for multivariate longitudinal data by assuming separate random effect on each outcome variable, and then combining them by imposing a joint multivariate distribution on the random effect. This approach has an advantage of having additional correlation emerging from the longitudinal data structure that can be modeled with the same frame-work, and compare the separate and joint models with respect to parameter estimation in longitudinal data analysis. To the best of our knowledge, no other study has been conducted to assess factors affecting jointly these two longitudinal inter-related outcomes (CD4 cell count & adherence to HAART) around the study area.

### Methods and data analysis

#### Study design

A retrospective cohort study was conducted to assess joint predictors of CD4 cell count change and adherence to HAART among adult HAART users enrolled in the first 10 months of 2008 and followed-up to June 2012. Joint modeling between count and ordinal responses was conducted.

#### Study area and population

The study was conducted at Felege-Hiwot Teaching and Specialized Hospital located in North-western Ethiopia, Amhara Region. The population of the study included HIV positive adults who initiated HAART treatment at Felege-Hiwot Teaching and Specialized Hospital.

#### Sample size and sampling procedure

From the total HIV positive adults who started HAART in the hospital from September 2008 to June 2012, 792 were selected using residence-based stratified random sampling technique. Patients with a minimum of 2 visits and a maximum of 23 visits were included in the study.

#### Data collection procedures

The study exclusively used secondary data. Therefore, a data extraction check-list was designed and used to adopt the routinely collected data. A baseline CD4 cell count data was identified and collected from the registration documents of HAART attendants. The first month adherence was also considered as covariate for longitudinal joint study. Similarly, other characteristics like socio-demographic variables, visiting times and clinical data were also collected from the registration documents of patients. The data were collected by health care service providers after they had been given adequate orientation about the variables included in the study.

### Data structure, compilation and analysis strategy

Secondary data were entered and analyzed using SAS version 9.2 software. For the sample to be included in the study, CD4 cell count measurement just before the initiation of HAART was considered as a covariate so that there could be at least two visit responses after the initiation of HAART for analysis.

#### Quality of data

The quality of the data was controlled by data controllers from ART section of the hospital. The controllers were taken intensive training by the Ministry of Health for different services. The data extraction tools and the variables included in the study were tested for consistency of understanding and the completeness of the data items on 45 random samples. Necessary amendments were made on the final data collection sheet.

#### Variables included in the study

The longitudinal response variables for current study were CD4 cell count change and adherence to HAART. The two response variables are different in nature. CD4 cell count change is defined as the difference between CD4 cells/mm^3^ in the current visiting time and CD4 cells/mm^3^ in visiting times immediately prior to the current response. Hence, $Y_{ij} - Y_{ij - 1}$, where *y*_*ij*_ is CD4 cell count result for the current visit and $Y_{ij - 1}$ is the CD4 cell count for the visit immediately prior to current visit which is discrete or count response. On the other hand, the adherence data obtained from the hospital is categorized as poor, fair and good adherence which is ordinal in nature, and measured with pills count. A patient is poor adherent if his/her performance is less than 85% of the prescribed medication; and he/she is a fair adherent if his/her performance is between 85 and 95% of the prescribed medication. If a patient’s adherence performance is at least 95% of the prescribed medication, such patient is categorized as a good adherent. The trend of adherence to HAART was tested using Cochran–Armitage test.

On the other hand, the predictor variables for the two responses were age in years, sex (male, female), marital status (living with partner, living without partner), ownership of cell phone (yes, no), weight in kilogram, baseline CD4 cell count in cells/mm^3^, disclosure of the disease to families (yes, no), residence area (rural, urban),WHO stages (stage I, stage II, stage III, stage IV), level of income (low, middle, high) visiting times (1, 2,…, 23) and first month adherence to HAART (poor, fair, good).

#### The impact of dropouts on the analysis

Patients who defaulted from HAART treatment will develop drug resistant virus and ultimately results in treatment failure and high risk of illness and death because of destruction of CD4 cell count by HIV. Non-adherence, therefore, reduces CD4 cell count; and this demands joint attention to maximally benefit from HAART. A logistic regression was conducted to assess whether or not missing values were affected by previous results; and this indicated that dropouts were independent of the previous outcomes ($\chi_{1}^{2}$ = 0.2018, p = 0.654). Dropout patients did not have reasons attributable to their progression rate of their previous visits; therefore dropout trend was Missed Completely at Random (MCAR). The trend of missing observation was assessed using a logistic regression model. Missing data were handled using multiple imputation technique.

A Chi square test of association and independent sampled t test were used for the comparison of baseline characteristics that will be included and excluded in the analysis for categorical and continuous variables, respectively. In model selection, we considered all predictors in the model, and fitted each product term obtained from predictor variables one at a time. This is important to assess the interaction effects of predictors on the variable of interest. A generalized linear mixed regression model (Quasi–Poisson) analysis was conducted to assess separate parametric estimation for the change of CD4 cell count [14]; and ordinal logistic regression was employed for separate data analysis of adherence to HAART [26]. The type of covariance structure and the magnitude of residual errors were also considered in model selection. In this regard, the model with the least within individual residual variability was selected.

#### Multivariate GLMM formulation of joint models

Since the specification of the joint distribution of the two responses is not straight forward, we can have two approaches for the formulation of joint multivariate models. The first approach is based on a conditioning argument that allows joint distribution to factor out in marginal and conditional component, where the conditioning can be done either on discrete or continuous outcome (avoiding direct specification of joint modeling) with introduction of probit approach. This approach does not directly lead to marginal inference, and the correlation between the two outcomes cannot be directly estimated. The second is direct formulation of joint modeling for both response variables with the introduction of Placket–Dale approach (Placket latent variable) assumption for modeling bivariate outcomes [27].

Instead of using a latent variable approach, one can directly specify the joint distribution for both outcomes through mixed model with specification of the marginal distribution, conditional on the correlated random effect. The generalized linear mixed model (GLMM) forms a very general class of subject-specific model which is used for univariate repeated measures. Joint model can be measured repeatedly over time or may be observed within a hierarchical trend. A GLMM can be easily adapted to situation where various outcomes are observed.

To obtain valid inferences, the joint model could account for the correction among the outcomes and effects of different factors. The joint generalized linear mixed model assumes that each outcome and the univariate models are combined through specification of joint multivariate distribution for all random effects. Furthermore, the mixed model can be applied with specification of marginal distribution, which is conditional on correlated random effect. To assess the association between CD4 cell count change and adherence to HAART for the data obtained at Felege-Hiwot Teaching and Specialized Hospital, the joint generalized linear mixed model was fitted. In this model, the correlation between the two responses is specified through the random effect structure assuming separate random intercept for each outcome and combining them by imposing joint multivariate distribution on the random intercept.

## Results

The baseline characteristics of patients included in the analysis indicates that the median age of patients was 36 years old (IQR 28–48). Of all the patients studied 50.6% were females, 40.1% were living in rural area, 55.2% were living without partners, 52.7% did not disclose the disease to family members living together and only 68.2% had good adherence in the first month treatment. Over 50% of the patients had attended their secondary education and over 50% of them had no cell phone. The average baseline CD4 cell count for all patients was 134 (IQR 113–180) and the average CD4 cell count change for the first month was 15.9 cell/mm^3^ (IQR 8–26). The average CD4 cell count change at all visits varies from 15.9 cells/mm^3^ at the 1st visit to 28 cells/mm^3^ at the 23rd visit. The corresponding standard deviations were 18 cells/mm^3^ at the first visit and 27 cells/mm^3^ at the last visit of the study period. Hence, the distribution at each visit shows that there were over-dispersion (variance > mean), and over-dispersed count response regression models should be considered for marginal analysis of CD4 cell count change data.

To fit the joint models of CD4 cell count change and adherence to HAART, first Quasi–Poisson for CD4 cell count change [14, 28] and ordinal logistic regression model for adherence to HAART data were considered separately [29]. Hence the parameter estimation for marginal models with log and cumulative logit link (Table 1) and conditional independence random-effect models (Table 2) were developed [30]. In addition, the parameter estimation for linear predictor or models which consist of the same variables already identified from GLMM models were developed (Table 3).

**Table 1:** Parameter estimates and corresponding standard errors of joint marginal model/separate analysis for CD4 cell count change data and adherence to HAART with un-structured working covariance

Parameter	CD4 cell count change			Adherence to HAART		
	Estimate	St. error	p value	Estimate	St. error	p value
Intercept	4.0066	0.0329	<0.0001	0.8921	0.2428	0.0002
Age	−0.0002	0.0007	0.0264*	−0.0111	0.0029	0.0001*
Weight	−0.0006	0.0002	0.0020*	0.0158	0.0027	<0.0001*
Initial CD4 cell count	0.0048	0.0002	<0.0001*	0.0410	0.0013	<0.0001*
Time	0.0238	0.0017	<0.0001*	−0.0429	0.0012	<0.0001*
Marital status (ref. = without part)						
Living with part	0.0064	0.0034	0.0464*	−0.0241	0.0481	0.6156
Sex (ref. = male)						
Female	−0.0131	0.0039	0.0009*	−0.4548	0.1036	<0.0001*
Area (ref. = urban)						
Rural	0.0213	0.0105	0.0425*	0.1517	0.1029	0.1405
Education (ref. = tertiary)						
No education	−0.0410	0.0059	<0.0001*	−0.0970	0.1480	0.5123
Primary education	−0.0406	0.0065	<0.0001*	0.0970	0.1646	0.5555
Secondary educ.	−0.0577	0.0055	<0.0001*	0.1135	0.1423	0.4248
Level of income (ref. = high income)						
Middle income	−0.0099	0.0054	0.0679	−0.1055	0.0772	0.1715
Low income	−0.0106	0.0041	0.0095*	0.1661	0.0572	0.0037*
Owner of cell phone (ref. = with phone)						
Without cell phone	−0.0204	0.0038	<0.0001*	0.6957	0.0975	<0.0001*
Level of disclosed the disease (ref. = yes)						
No	−0.0062	0.0034	0.0715	0.2834	0.0484	<0.0001*
WHO stages (ref. = stage IV)						
Stage I	0.1191	0.0077	<0.0001*	−0.1978	0.1101	0.0725
Stage II	0.1239	0.0064	<0.0001*	0.2195	0.0904	0.0152*
Stage III	0.1006	0.0063	<0.0001*	0.1500	0.0886	0.0904
Time * ownership of cell phone (ref. = yes)						
Time * no	−0.0099	0.0013	<0.0001*	−0.0094	0.0077	0.2236
Time * area (ref. = urban)						
Time * rural	−0.0024	0.0020	0.2192	0.0033	0.0082	0.2236
Time * sex (ref. = male)						
Time * female	0.0027	0.0006	<0.0001*	0.0226	0.0067	0.0007*
Time * education (ref. = tertiary)						
Time * no educ.	0.0062	0.0007	<0.0001*	0.0300	0.0114	0.0085*
Time * primary educ.	0.0086	0.0008	<0.0001*	0.0315	0.0128	0.0138*
Time * secondary educ.	0.0067	0.0007	<0.0001*	0.0376	0.0111	0.0007*
Age * sex (ref. = male)						
Age * female	−0.0046	0.0004	<0.0001*	0.0078	0.0081	0.0345*
Age * education (ref. = tertiary)						
Age * no educ.	−0.0045	0.0006	<0.0001*	−0.0752	0.0098	<0.0001*
Age * primary educ.	−0.0022	0.0007	0.0015*	−0.1197	0.0115	<0.0001*
Age * secondary educ.	−0.0036	0.0006	<0.0001*	−0.0501	0.0102	<0.0001*
Age * area (ref. = urban)						
Age * rural area	−0.0025	0.0004	<0.0001*	0.0014	0.0065	0.8275
Mar. stat * level of exposedness (ref. = closed)						
Mar. stat * disclosed the disease	0.0067	0.0007	0.0652	0.2001	0.0989	0.0430*

* Significant p values

**Table 2:** Parameter estimates and corresponding standard errors for conditional independence random intercept model with CD4 cell count change data and adherence to HAART

Parameter	CD4 cell count change			Adherence to HAART		
	Estimate	St. error	p value	Estimate	St. error	p value
Intercept	4.0066	0.0329	<0.0001	0.8921	0.2428	0.0002*
Age	−0.0002	0.0007	0.0264*	−0.0111	0.0029	0.0001*
Weight	−0.0006	0.0002	0.0020*	0.0158	0.0027	<0.0001*
Initial CD4 cell count	0.0048	0.0002	<0.0001*	0.0410	0.0013	<0.0001*
Time	0.0238	0.0017	<0.0001*	0.0129	0.0312	<0.0001*
Marital status (ref. = without part)						
Living with part	0.0064	0.0034	0.0464*	−0.0241	0.0481	0.6156
Sex (ref. = male)						
Female	−0.0131	0.0039	0.0009*	−0.4548	0.1036	<0.0001*
Area (ref. = urban)						
Rural	0.0213	0.0105	0.0425*	0.1517	0.1029	0.1405
Education (ref. = tertiary)						
No education	−0.0410	0.0059	<0.0001*	−0.0970	0.1480	0.5123
Primary education	−0.0406	0.0065	<0.0001*	0.0970	0.1646	0.5555
Secondary educ.	−0.0577	0.0055	<0.0001*	0.1135	0.1423	0.4248
Level of income (ref. = high income)						
Middle income	−0.0099	0.0054	0.0679	−0.1055	0.0772	0.1715
Low income	−0.0106	0.0041	0.0095*	0.1661	0.0572	0.0037*
Owner of cell phone (ref. = with phone)						
Without cell phone	−0.0204	0.0038	<0.0001*	0.6957	0.0975	<0.0001*
Level of disclosure (ref. = disclosed)						
Closed the disease	−0.0062	0.0034	0.0715	0.2834	0.0484	<0.0001*
WHO stages (ref. = stage IV)						
Stage I	0.1191	0.0077	<0.0001*	−0.1978	0.1101	0.0725
Stage II	0.1239	0.0064	<0.0001*	0.2195	0.0904	0.0152*
Stage III	0.1006	0.0063	<0.0001*	0.1500	0.0886	0.0904
Time * ownership of cell phone (ref. = yes)						
Time * no	−0.0099	0.0013	<0.0001*	−0.0094	0.0077	0.0236*
Time * area (ref. = urban)						
Time * rural	−0.0024	0.0020	0.2192	0.0033	0.0082	0.0236*
Time * sex (ref. = male)						
Time * female	0.0027	0.0006	<0.0001*	0.0226	0.0067	0.0007*
Time * education (ref. = tertiary)						
Time * no educ.	0.0062	0.0007	<0.0001*	0.0300	0.0114	0.0085*
Time * primary educ.	0.0086	0.0008	<0.0001*	0.0315	0.0128	0.0138*
Time * secondary educ.	0.0067	0.0007	<0.0001*	0.0376	0.0111	0.0007*
Age * sex (ref. = male)						
Age * female	−0.0046	0.0004	<0.0001*	0.0078	0.0081	0.0345*
Age * education (ref. = tertiary)						
Age * no educ.	−0.0045	0.0006	<0.0001*	−0.0752	0.0098	<0.0001*
Age * primary educ.	−0.0022	0.0007	0.0015*	−0.1197	0.0115	<0.0001*
Age * secondary educ.	−0.0036	0.0006	<0.0001*	−0.0501	0.0102	<0.0001*
Age * area (ref. = urban)						
Age * rural area	−0.0025	0.0004	<0.0001*	0.0014	0.0065	0.8275
Mar. stat * level of exposedness (ref. = closed)						
Marital stat * disclosed the disease	0.0067	0.0007	0.0652	0.2001	0.0989	0.0430*

* Significant p values

**Table 3:** Parameter estimates for joint model of CD4 cell count change data using linear predictor

Parameter	Estimate	St. error	Adjusted rate ratio (ARR)	Wald 95% CI		p value
Intercept	4.0066	0.0029	54.9596	51.5318	58.6156	<0.0001
Age	−3.4201	0.0001	0.0327	0.0098	0.0899	0.0264*
Weight	0.0036	0.0001	1.0036	0.0021	1.0045	0.0820
Baseline CD4 cell count	0.0048	0.0002	1.0048	1.0035	1.0061	<0.0001*
Time	0.0238	0.0007	1.0240	1.0206	1.0277	<0.0001*
Marital status (ref. = without part)						
Patients living with partner	0.0064	0.0014	1.0064	0.9998	1.0132	0.0564
Sex (ref. = male)						
Female	0.0131	0.0005	1.0132	0.0124	1.0155	0.0659
Area (ref. = urban)						
Rural	−2.65	0.0005	0.0706	0.9558	1.0982	0.0525
Education (ref. = tertiary)						
No educ.	−4.02	0.0019	0.0183	0.9487	1.0298	0.0627
Primary educ.	−3.45	0.0005	0.0317	0.9480	1.9725	0.0801
Secondary educ.	−2.86	0.0015	0.0573	0.9337	1.0481	0.0568
Level of income (ref. = high income)						
Middle income	−4.12	0.0014	0.0162	0.9797	1.0007	0.0679
Low income	−3.63	0.0001	0.0265	0.9816	1.0974	0.0095
Owner of cell phone (ref. = yes)						
No	−3.54	0.0018	0.0290	0.0172	0.0870	<0.0001*
First month adherence level (ref. = good adherence)						
Fair adherence	−3.54	0.0002	0.0290	0.0097	0.0439	<0.0001*
Poor adherence	−2.86	0.0091	0.0573	0.0214	0.0706	<0.0001*
Level of disclosed the disease (ref. = yes)						
No	−2.6	0.0014	0.0743	0.9871	1.0995	0.0715
WHO stages (ref. = stage IV)						
Stage I	0.1191	0.0007	1.1265	0.1097	1.1436	0.0651
Stage II	0.1239	0.0014	1.1319	0.1173	1.0461	0.0612
Stage III	0.1006	0.0023	1.1058	0.0923	1.0196	0.0851
Time * owner of cell phone (ref. = yes)						
Time * no	−2.96	0.0003	0.0518	0.0443	0.0996	<0.0001*
Time * area (ref. = urban)						
Time * rural	−2.4	0.0010	0.0907	0.9937	1.0004	0.0792
Time * sex (ref. = male)						
Time * female	0.0027	0.0002	1.0027	1.0016	1.0038	<0.0001*
Time * education (ref. = tertiary)						
Time * no educ.	−4.00	0.0007	0.0183	0.0082	0.0452	<0.0001*
Time * primary educ.	−3.83	0.0002	0.0124	0.0044	0.0360	<0.0001*
Time * secondary educ.	−3.43	0.0001	0.0086	0.0053	0.0147	<0.0001*
Age * sex (ref. = male)						
Age * female	−3.421	0.0002	0.0326	0.0144	0.0662	<0.0001*
Age * education (ref. = tertiary)						
Age * no educ.	0.042	0.0002	1.0433	1.0126	1.0799	<0.0001*
Age * primary educ.	0.032	0.0001	1.0325	1.0112	1.0601	0.0015*
Age * secondary educ.	0.023	0.0004	1.0232	1.0093	1.0594	<0.0001*
Age * area (ref. = urban)						
Age * rural area	0.0025	0.0002	1.0025	0.0017	1.0034	0.08621

* Significant at 5% level of confidence for joint model

Table 1 indicates the separate or joint marginal models for CD4 cell count change and adherence to HAART using Quasi–Poisson regression and ordinal logistic regression. As indicated in the Table 1, age, weight, initial CD4 cell count and ownership of cell phone significantly affected both outcomes. From the two-way interaction, time * level of education, time * sex, age * sex as well as age * level of education were significant for both outcome variables. The separate models shown in Table 1 were univariate distributions. Table 2, on the other hand, shows the combination of the separate models by imposing joint multivariate distribution on the random effect. The analysis was done using generalized log likelihood function with Laplace approximation. The conditional independence of random-effect models considered in this analysis shows that the GLMM approach can be extended to multivariate longitudinal data by assuming separate random effect for each outcome and combining them by imposing a joint multivariate distribution on the random effects. The SAS procedure using general log-likelihood function allows one to impose the joint multivariate distribution on the random effects from the two separate models. The results obtained from fitting a joint model for the two response variables of uncorrelated random intercept using the GLIMMIX procedure were used as initial parameter estimates.

Table 2 shows the conditional independence random intercept model. As indicated in the Table 2, patients’ age, weight, initial CD4 cell count, visiting time, ownership of cell phone and sex were jointly and significantly associated with both response variables. The same sign in parametric estimation indicates that the two outcomes are positively correlated to each other. Since the conditional independence assumption might be too restrictive, we, therefore, attempted to relax the conditional independence assumption by re-fitting the joint random intercepts model with possible correlated errors. However, the conditional independence approach, which we attempted, failed to converge the model. During this time, introducing conditional dependence of one response in terms of the other using linear predictor is important [31]. This approach is also helpful to validate the observed correlation between the two outcomes emerging from the association of the random intercepts. We fitted a generalized linear mixed model for CD4 cell count change as response variable including adherence to HAART in the linear predictor. The result is presented in Table 3 below. The result indicates that CD4 cell count change is positively correlated (p ≤ 0.0001) with adherence to HAART.

As shown in Table 3, age, weight, baseline CD4 cell count, visiting times, marital status, sex, residence area, ownership of cell phone, disclosure of the disease to family members and first month adherence have significantly affected CD4 cell count change. Hence, as age of a patient increased by 1 year, the log of change of CD4 cell count decreased by 3.3% cell/mm^3^ keeping the other variables constant [ARR = 0.0327, 95% CI (0.0098, 0.0899); p = 0.0264]. As the number of visiting times increased by one unit, the log of change of CD4 cell count increased by 2.4% [ARR = 1.0240, 95% CI (1.0206, 1.0276); p < 0.0001]. The analysis revealed that the rate of change of CD4 cell count for patients without ownership of cell phone was 2.9% less as compared to patients with ownership of cell phone [ARR = 0.0290, 95% CI (0.0172, 0.0870); p < 0.0001]. The log of change of CD4 cell count for patients with fair adherence at a given visiting time was 2.9% less than those patients who had good adherence [ARR = 0.0290133, 95% CI (0.00974, 0.043931); p < 0.0001] and the log of change of CD4 cell count for patients with poor adherence at visiting time, t was 5.7% less than those of good adherent patients [ARR = 0.0573, 95% CI (0.0214, 0.0706); p < 0.0001]. From the two way interaction effects; time * ownership of cell phone, time * sex, time * level of education, age * sex, age * level of education were significantly and jointly affected both responses through linear link (refer Table 3).

### Interaction effects of ownership of cell phone and visiting times

**Fig. 1:**
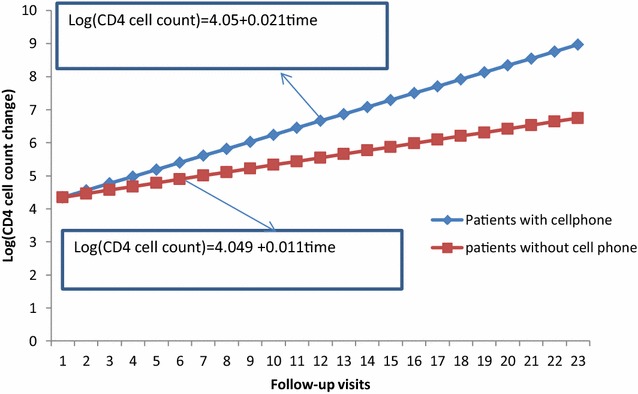
Interaction plot between ownership of cell phone and follow-up visits

For a unit increase of visiting time, the rate of change of CD4 cell count for patients without owner of cell phone was 5.2% less than patients with owner of cell phone [ARR = 0.0518, 95% CI (0.0443, 0.0996); p < 0.0001]. Figure 1 indicates that the log of change of CD4 cell count for patients with owner of cell phone was by far better than those patients without cell phone as visiting time increased.

### Interaction effect between sex of patients and follow-up visits

**Fig. 2:**
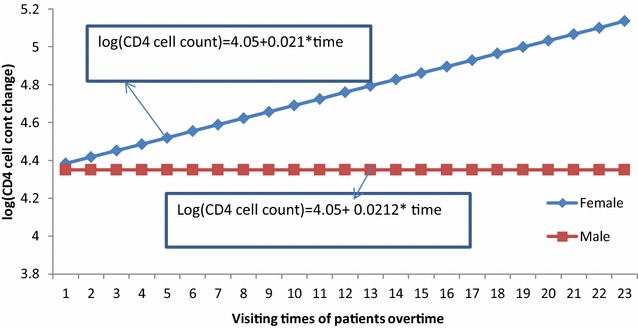
Interaction plot between sex of patients and their follow-ups

As visiting times of adult patients increased by one unit, the log of change of CD4 cell count for female patients was 0.3% greater than male patients [ARR = 1.0027, 95% CI (1.0016, 1.0038); p < 0.0001]. From Fig. 2, we saw that as visiting time increased, the log of change of CD4 cell count for female patients is greater than that of male patients.

### Interaction effect between age and sex of patients

**Fig. 3:**
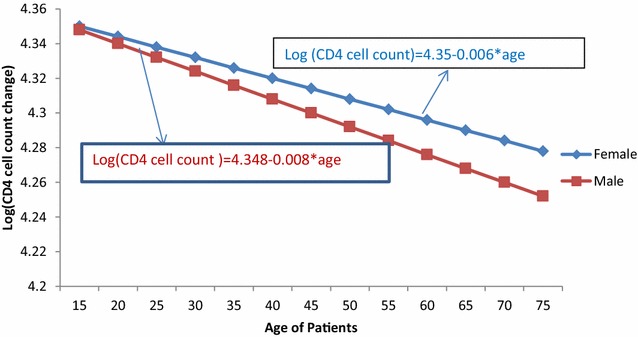
Interaction plot between sex and age of patients

From Table 3, it is indicated that as age of patients increased by 1 year, the decreasing rate of change of CD4 cell count for female patients was 3.3% less than male patients [ARR = 0.0326, 95% CI (0.0144, 0.0662); p < 0.0001]. Hence, as age of patients increased, the decreasing rate for female patients was less probable as compared to male patients (refer to Fig. 3).

### Interaction effect between level of education and visiting times

**Fig. 4:**
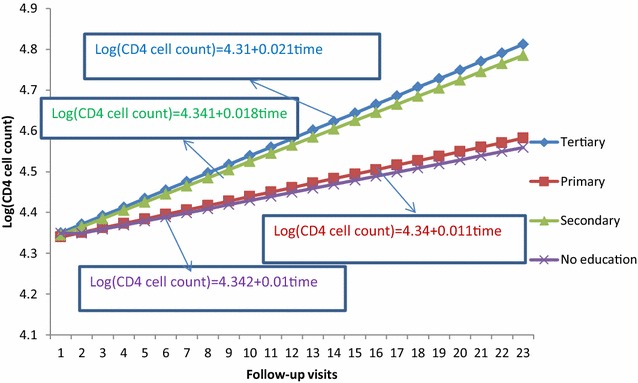
Interaction plot between level of education and follow-up visits

As visiting time of patients increased by one unit, the rate of change of CD4 cell count for non-educated patients was 1.8% less than those of tertiary educated patients [ARR = 0.0183, 95% CI (0.0082, 0.0452); p < 0.0001]. Similarly comparing primary educated patients with tertiary level, the log of change of CD4 cell count for primary educated patients was 1.2% less than tertiary levels [ARR = 0.0124, 95% CI (0.0044, 0.03662); p < 0.0001] and the log of change of CD4 cell count for secondary educated patients was 0.8% less than tertiary educated patients [ARR = 0.0086, 95% CI (0.0053, 0.0147); p < 0.0001] (refer to Table 3). Figure 4 indicates that as visiting time of patients increased, patients with tertiary education perform high log of CD4 cell count change as compared to the other group.

### Interaction effect between level of education and age of patients

**Fig. 5:**
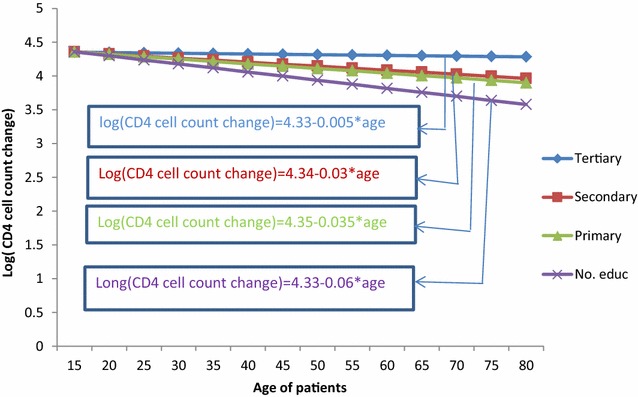
Interaction plot between level of education and age of patients

It is known from the literature that as age of patients increase, the log of change of CD4 cell count decrease. However, the decreasing rate for different level of education was different from the current analysis. Hence as age of a patient increased by 1 year, the decreasing rate of CD4 cell count for non-educated patient was 4.3% greater as compared to tertiary educated patients [ARR = 1.0433, 95% CI (1.0126, 1.0799); p < 0.0001]; the log of change CD4 cell count for primary educated patients was 3.2% greater than tertiary educated patients [ARR = 1.0325, 95% CI (1.0112, 1.0601); p = 0.0015]; and decreasing rate of CD4 cell count for secondary educated patients was 2.3% greater than tertiary educated patients [ARR = 1.0232, 95% CI (1.0093, 0.0594); p < 0.0001]. Figure 5 indicates that as age of patients increase, the decreasing rate of tertiary educated patients was lower than other groups.

## Discussion

We have presented a general approach to the joint analysis of two longitudinal response variables assuming their separate analysis. The results in this study indicated that the separate models which did not include patients’ specific effects were not significantly different from joint models developed with the assumption of separate analysis. As indicated above the joint models were formed by imposing the joint multivariate distribution of random effect, Hence, the results of both separate and joint analysis were consistent. However, the joint models were simpler as compared to the separate models as their effective member of parameters was smaller. In other words, the reduction of the number of effective parameters ensures that joint models were more parsimonious/less complex in parameter estimation of longitudinal data analysis. This result complies with previous researches [30, 32, 33]. Furthermore, joint models were advantageous for answering multivariate questions at the same time. They also helped assess the correlation between the two response variables and gave ample opportunity to see predictors of the two response variables jointly.

The result in this study indicated that CD4 cell count change and adherence to HAART had been influenced jointly by some of the covariates like age, weight, baseline CD4 cell count, visiting times, sex, ownership of cell phone, first month adherence and level of disclosing the disease to family members and by their interaction effects (refer to Table 3). Recent findings from longitudinal study [34] also proved that CD4 cell count change was affected by many of these covariates. This finding suggests that the hospital should advise adult HIV patients to adhere the prescribed medication on time to achieve high CD4 cell count change. Patients, who had cell phone, had more probability to adhere the prescribed medication than patients who had not. This result is supported by another study [17]. Hence alarm of cell phone/memory aids/can be used to remember patients the time for taking pills and visiting health institutions.

As ages of patients increase, CD4 cell count and adherence to HAART decrease. But, the decreasing rate of these response variables for patients with tertiary education is less likely as compared to that of the others. This might occur because as patients become more educated, they may have better care of their health. This result has been supported by previous study [16]. Patients with high initial CD4 cell count adhere the prescribed medication more; and these results in high rate of change of CD4 cell count through time. This result suggests that patients should start their HAART without keeping the cut-off points (200 cell/mm^3^). This result is supported by previous study [35] on the impact of adherence on CD4 cell count change.

The two responses were higher for urban adult patients than rural areas through time. The reason for this might be the long distance of the rural areas from health institutions, and patients in these areas may come after high destruction of CD4 cells by HIV. There are discordant other findings concerning this. For instance, the result of this study agrees with a previous study [36]. However, another study indicates that rural patients’ adherence to HAART is better through time, and this implies that they have better CD4 cell count [37]. Therefore, this issue needs further investigation for reliable information.

Patients who lived with a partner had more probability to adhere the prescribed medication and had high CD4 cell count change as compared to patients who lived without partners. This can be associated with lack of social support for patients living without partner. This result had been supported by former research [17]. The result of this study also indicates that female patients had more probability to adhere the prescribed medication than males, and through time they had achieved high rate of change of CD4 cell count. This result, however, is inconsistent with previous studies [38].

### Limitations

The interactions between variables, which were not expected during the data collection process, were identified during data analysis. The cause for these interaction occurrences was not detected and explained. Adherence to HAART, which was one of the response variables, was measured in pill count technique. This technique had a disadvantage that include patients’ switching of medicines between bottles and discarding pills before visits [27]. Despite its limitations, pill count technique has strong linear relationship with viral load [39]. The result in this analysis is true only for adult patients; that is, it may have different outcomes when patients with all ages are considered. This could be a potential area for further investigation.

## Conclusions

Longitudinal joint models fitted for the joint data analysis under this investigation. Generalized linear mixed model was extended to multivariate cases in the data analysis process. This had been done using marginal/separate analysis as well as conditional independent random effect models and a random intercept model with correlated residual error structure. Developing joint models has helped validate the observed correlation between the outcomes emerging from the association of intercepts. The result under this investigation indicated that some covariates were significantly associated with the change of CD4 cell count; and the development of joint model for the two responses gave powerful estimation as compared to the separate or marginal model. The result of this study identified a certain group of patients who were with maximum risk of CD4 cell count change, but who had poor adherence performance. This group of patients needs high intervention in counseling and awareness creation.

## Abbreviations

AIC: Akaike Information Criteria
BIC: Bayesian Information Criteria
CI: confidence interval
CD4: classification determinant four
HAART: highly active anti-retroviral therapy
HIV: human immune deficiency virus
WHO: World Health Organization
MLE: maximum likelihood estimator
GLM: generalized linear model
GLMMs: generalized linear mixed modes
MCAR: missed completely at random
ARR: adjusted rate ratio
IQR: inter quartile range
